# Supplementary figures and images for: Unravelling the MicroRNA-Mediated Gene Regulation in Developing Pongamia Seeds by High-Throughput Small RNA Profiling
Source: Int J Mol Sci. 2019 Jul 17;20(14):3509. doi: 10.3390/ijms20143509 (PMC6678122; doi:10.3390/ijms20143509)

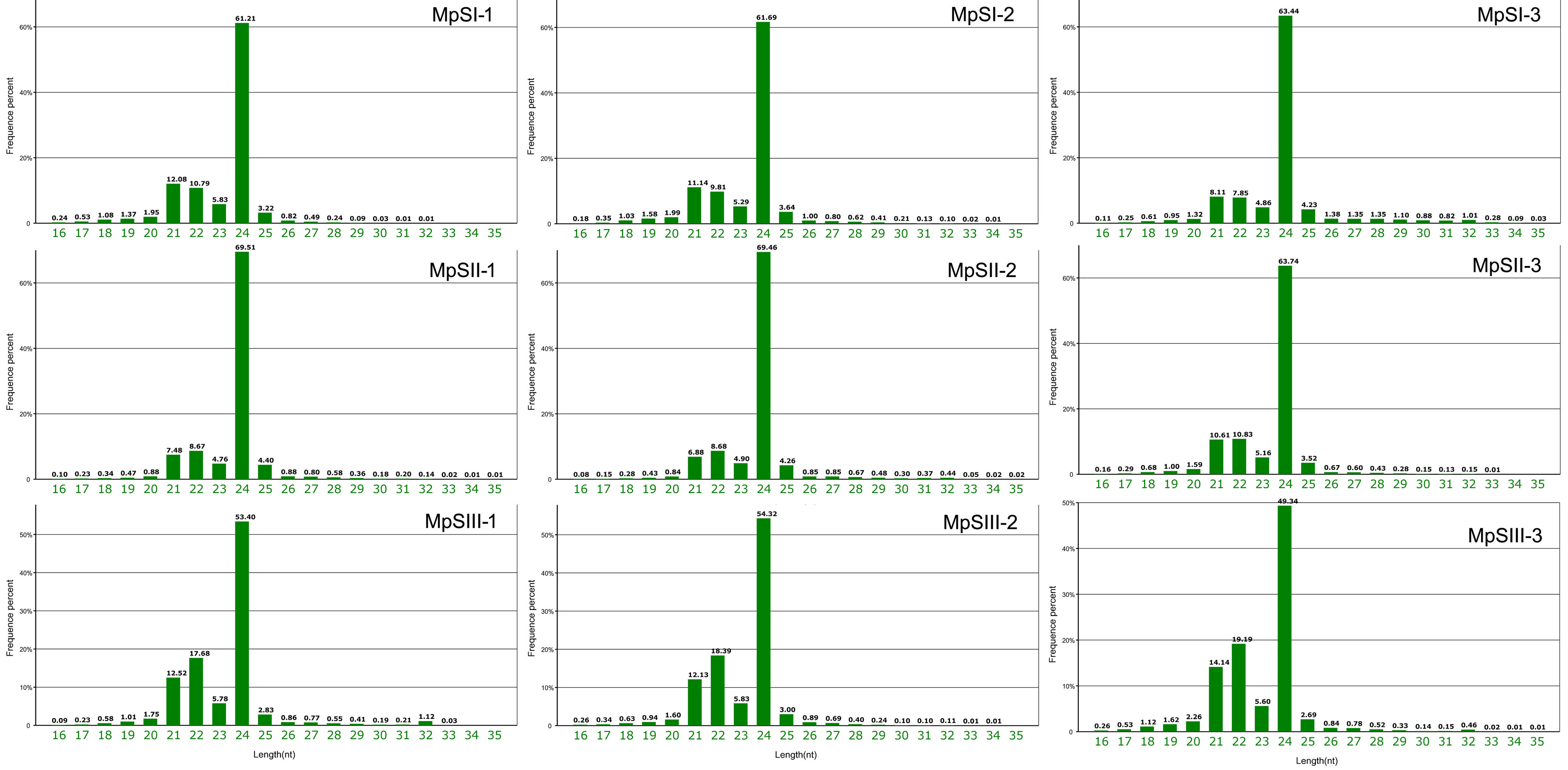

Supplement: Supplementary file 1 [file ijms-20-03509-s001.zip › Supplementary Files/Figure S1.jpg]

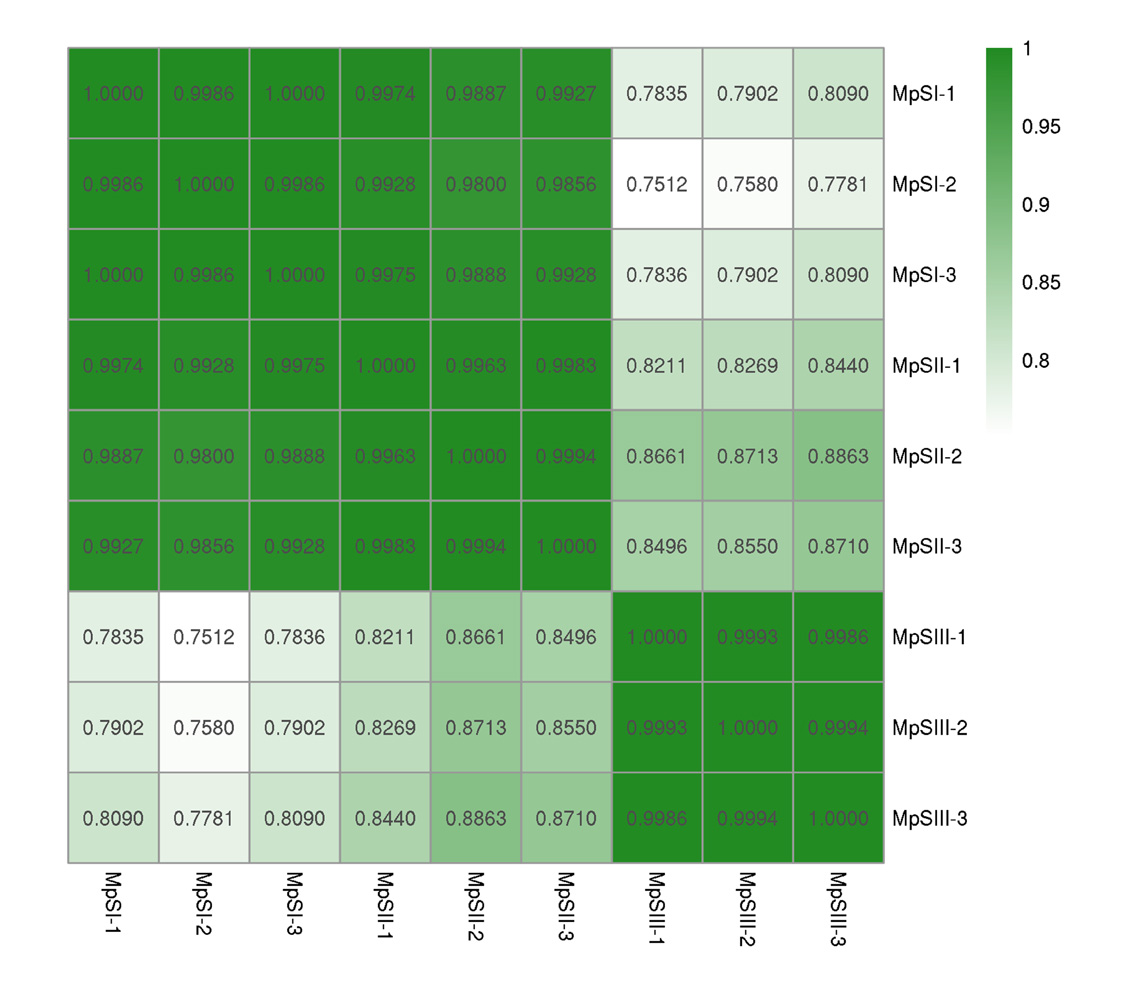

Supplement: Supplementary file 1 [file ijms-20-03509-s001.zip › Supplementary Files/Figure S2.jpg]

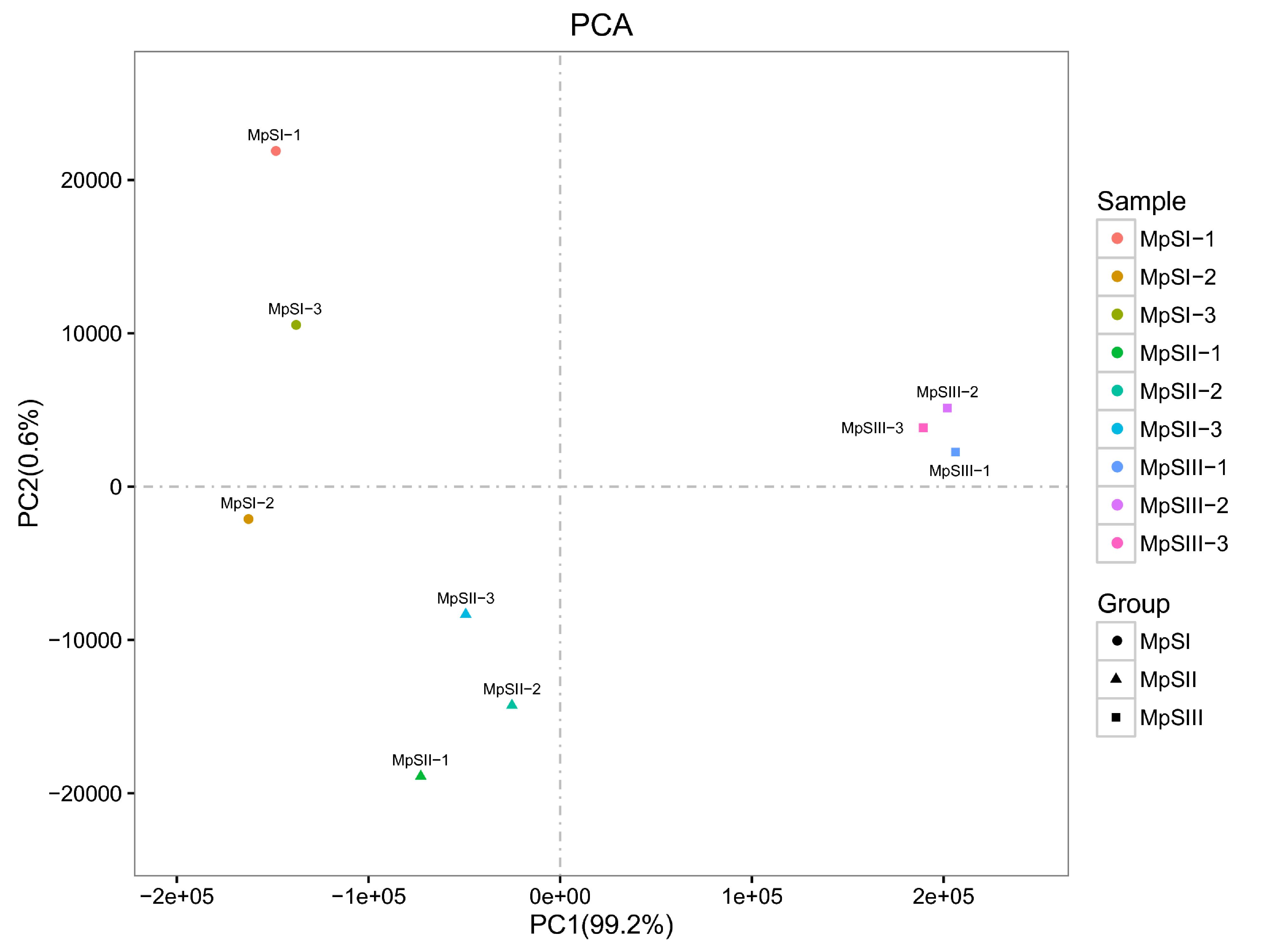

Supplement: Supplementary file 1 [file ijms-20-03509-s001.zip › Supplementary Files/Figure S3.jpg]

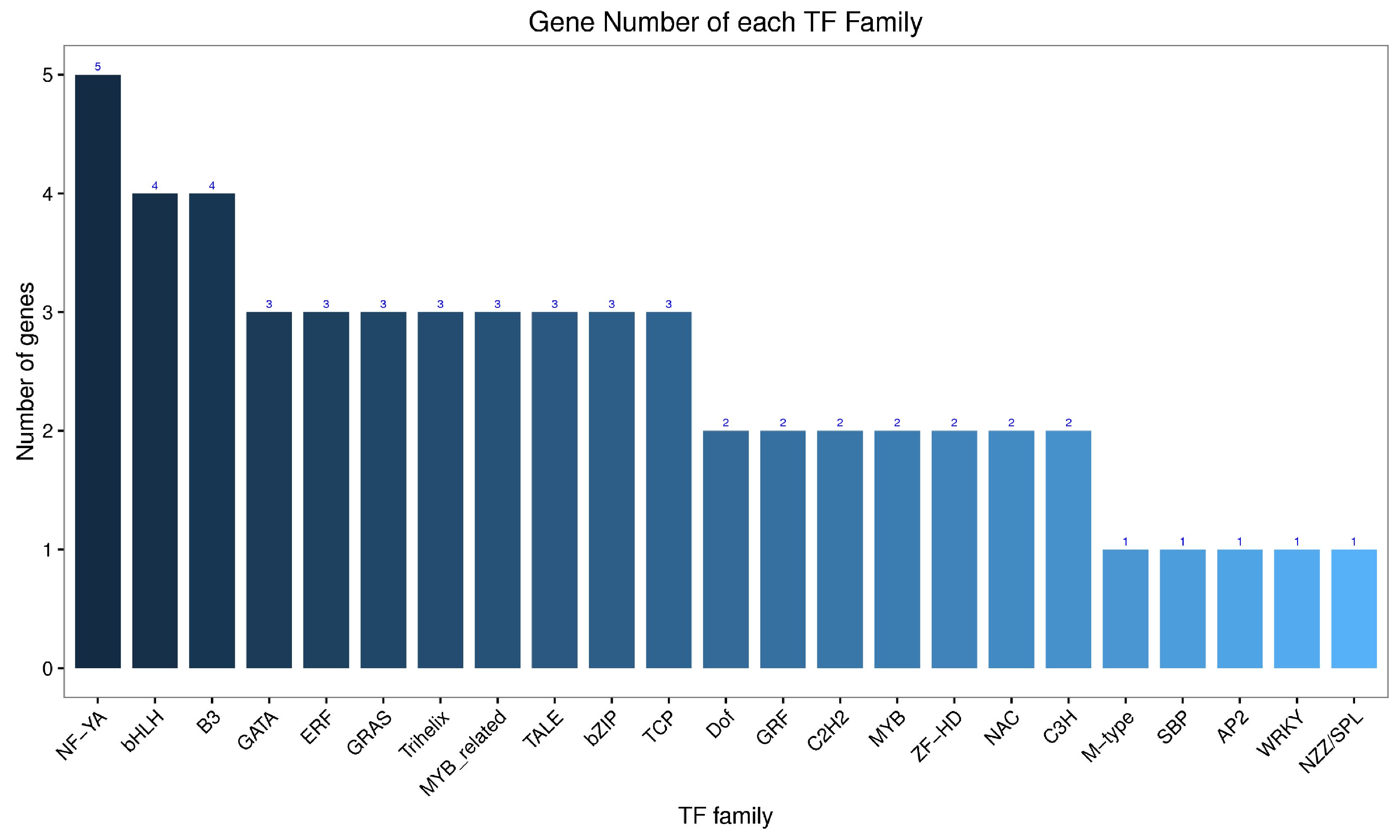

Supplement: Supplementary file 1 [file ijms-20-03509-s001.zip › Supplementary Files/Figure S4.jpg]

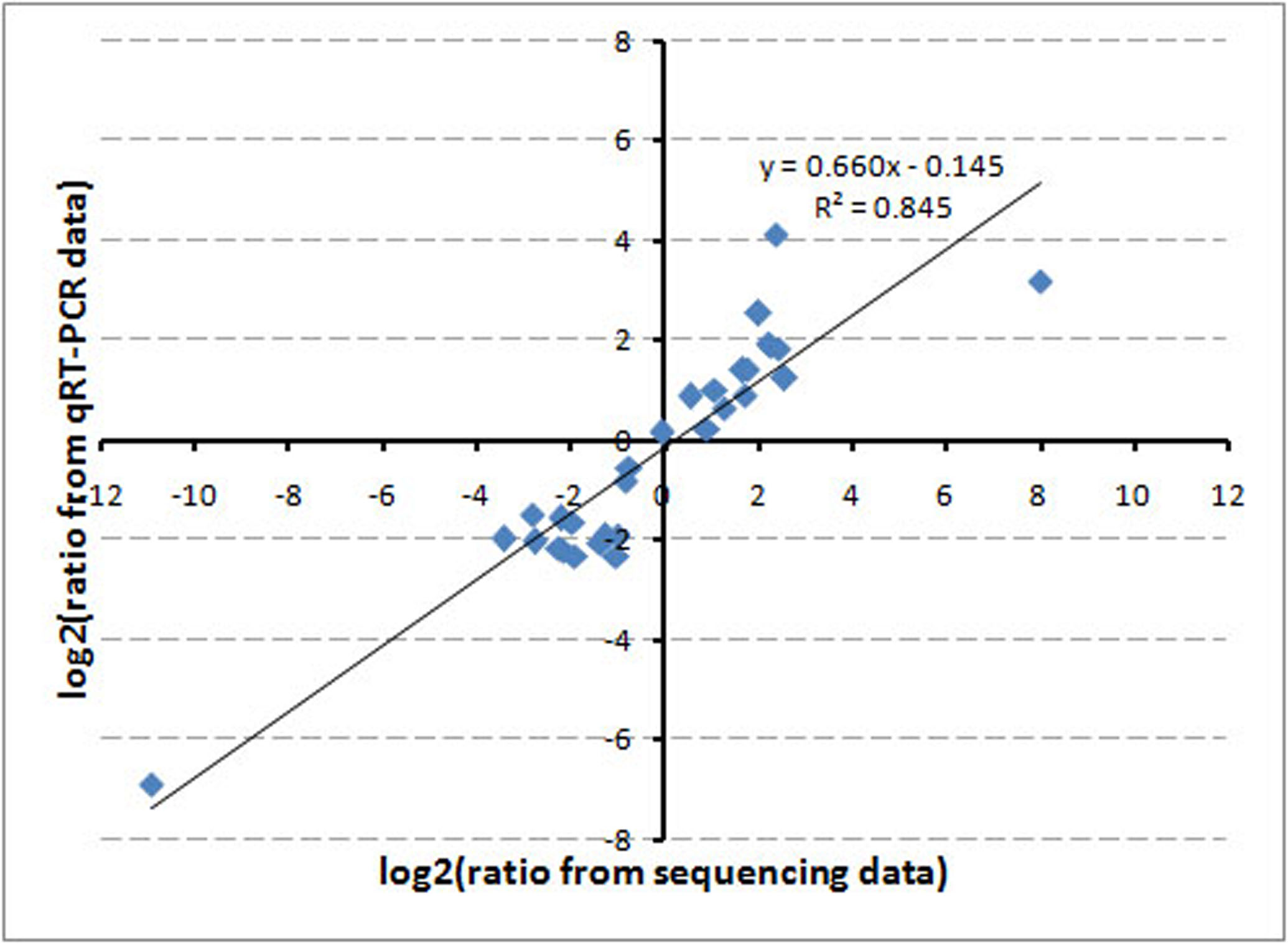

Supplement: Supplementary file 1 [file ijms-20-03509-s001.zip › Supplementary Files/Figure S5.jpg]

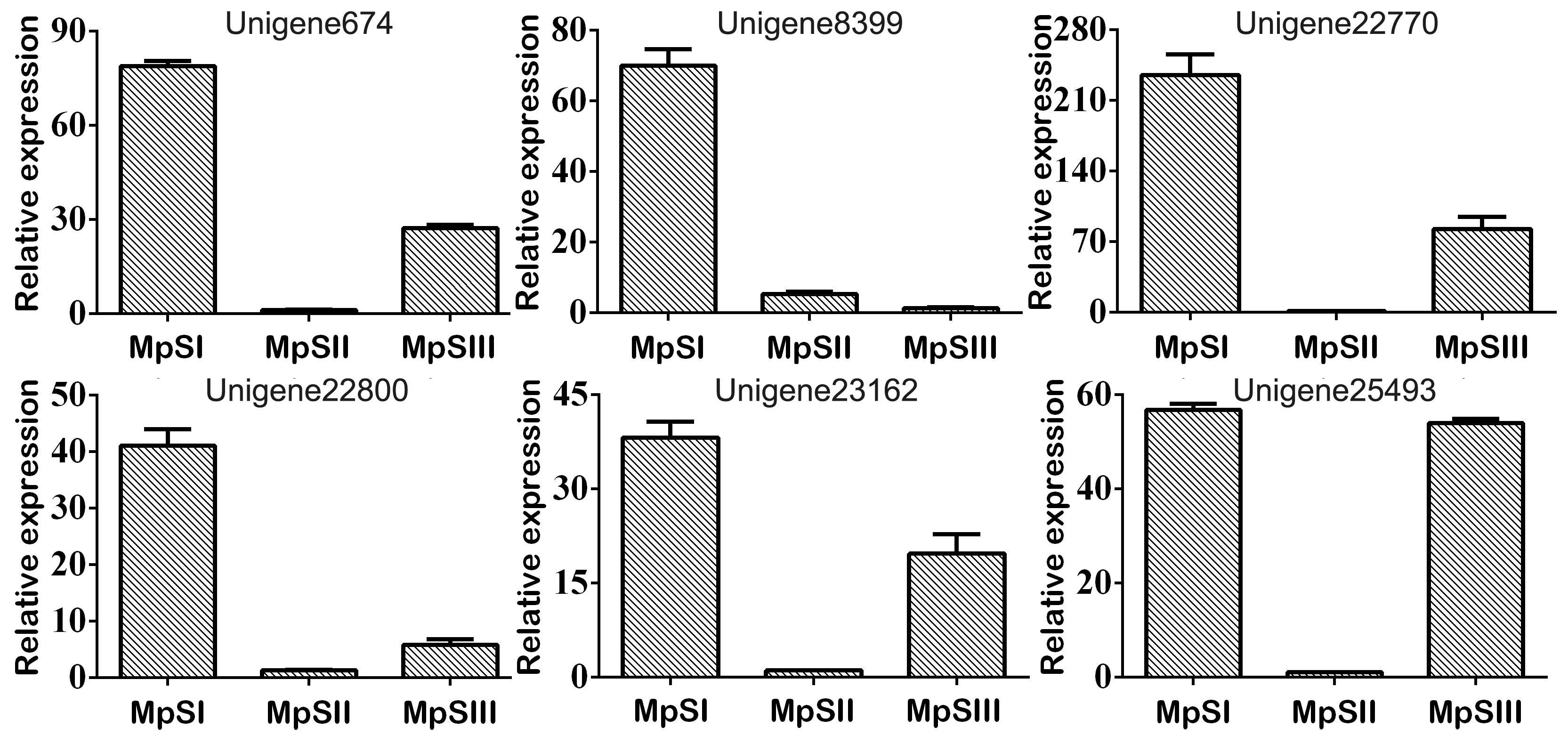

Supplement: Supplementary file 1 [file ijms-20-03509-s001.zip › Supplementary Files/Figure S6.jpg]
